# Orbital dermatofibrosarcoma protuberans with intracranial extension preceded by recurrent leiomyoma of the orbit: a case report

**DOI:** 10.1186/s13256-015-0561-4

**Published:** 2015-04-29

**Authors:** Sanaullah Bashir, Maryam Tariq, Hafiz Muhammad Aslam, Abdul Sattar M Hashmi, Baber Malik, Akhtar Amin, Sidra Mumtaz

**Affiliations:** 1 Dow Medical College, Dow University of Health Sciences, Karachi, Pakistan; 2 Department of Neurosurgery, Jinnah Postgraduate Medical Center, Karachi, Pakistan; 3 Department of Molecular Diagnostic and Immunology Laboratory, Sindh Institute of Urology and Transplantation, Aga Khan University, Karachi, Pakistan

**Keywords:** Dermatofibrosarcoma protuberans, Leiomyoma, Orbital tumors, Soft tissue tumors

## Abstract

**Introduction:**

Dermatofibrosarcoma protuberans is a rare, locally aggressive cutaneous tumor of intermediate to low-grade malignancy. *COL1A1-PDGFβ* translocation is specific to dermatofibrosarcoma protuberans, where the abnormally fused *COL1A1-PDGFβ* gene directs formation of an abnormal combined (fusion) protein that researchers believe to ultimately function like the platelet-derived growth factor-beta protein.

**Case presentation:**

In this report, we present a case of a 63-year-old Asian man with dermatofibrosarcoma protuberans of the right orbit with intracranial extension. He had a prior history of recurrent leiomyomas at the identical site. He underwent near-total *en bloc* resection of the tumor through a wide craniectomy with a 6cm rim of the frontal scalp, allowing the tumor to be resected *en bloc*, leaving negative margins. Microscopically, the tumor comprised spindle cells with mild nuclear atypia and a low mitotic index embedded in a spiraling pattern of decussating fascicles consistent with dermatofibrosarcoma protuberans. The lesion was positive for CD34 and BCL2. Following resection, the patient was started on imatinib mesylate therapy (800mg/day).

**Conclusions:**

We propose that platelet-derived growth factor, which has been implicated in the progression of leiomyomas by augmenting mitogenesis, may have acted in an autocrine manner to cause cell division, which may have led to the development of dermatofibrosarcoma protuberans in our patient. Further research is imperative to find certain molecular associations between the discussed soft tissue tumors. Also important is the effective utilization of platelet-derived growth factor receptor kinase inhibitors to prevent transformation to any platelet-derived growth factor–driven tumor, which in our patient was a dermatofibrosarcoma protuberans.

## Introduction

Dermatofibrosarcoma protuberans (DFSP) is a rare spindle cell tumor [1]. It accounts for less than 0.1% of all the malignant lesions and only 1% of all soft tissue sarcomas [2,3]. It is a cutaneous malignant neoplasm that originates from the dermis [1] and extends into the subcutaneous tissues and muscles [4]. Men are more frequently affected than women. It occurs in all age groups, most commonly in the second to fifth decades of life [5]. No exogenous risk factors are known to account for DFSP, nor is there evidence of genetic inheritance. However, studies have shown a reciprocal translocation between chromosomes 17 and 22 (17q22; 22q13) in more than 90% of DFSP tumor cells.

Orbital leiomyoma is another rare tumor. In 2004, Gündüz and coworkers reported 16 cases of orbital leiomyoma documented in the past 40 years, with men being predominantly affected [6]. Most of the tumors have been deeply located within the orbit, with a slight predilection for the inferior and medial quadrants [7].

Here we present a case of a patient with a DFSP that was preceded by recurrent orbital leiomyoma. This makes it significantly rare because both the uncommon forms of tumor occurred in the same individual and, more remarkably, at almost the same location—the right orbit.

## Case presentation

A 63-year-old Asian man of low socioeconomic status presented to our institution with a complaint of a 1-year history of progressively enlarging swelling on the anteromedial aspect of the right orbit that was causing proptosis and complete vision loss of the ipsilateral eye. He had a significant medical history of diabetes mellitus. He had no constitutional or neurological symptoms and no signs evocative of other sites of involvement. He had no family history of cancer, smoking, intravenous drug abuse or sexual promiscuity.

**Figure 1:**
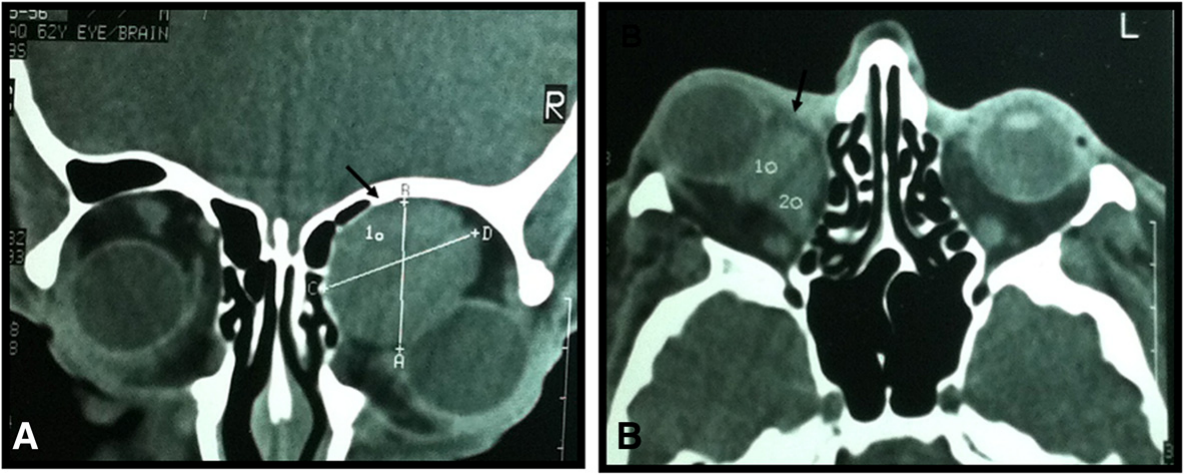
Orbital computed tomographic scans showing leiomyoma. **(A)** Coronal image. **(B)** Axial image. These computed tomographic scans reveal a well-defined soft tissue mass in the anterosuperior aspect of the right orbit (arrows in **A** and **B**). The mass is displacing the eyeball anteroinferiorly **(A)**. The lesion reveals mild enhancement with intravenous contrast dye and is displacing the medial rectus muscle laterally **(B)**. The left eyeball and rectobulbar spaces are normal.

Eight years earlier, he had developed right orbital swelling causing severe proptosis, diminished vision and restricted eye movements. Computed tomography (CT) revealed an extraconal mass measuring 3.3cm×3cm that was causing erosion of the superior margin of the orbit with right frontal sinus extension (Figures 1). An excisional biopsy was done via the transcranial route, and the specimen was sent for histopathological examination. Grossly, the tumor had a solid, lobulated appearance. Microscopically, it consisted of bundles of spindle-shaped cells with oval nuclei that had blunted ends interspersed in fibrous stoma. Immunohistochemistry demonstrated that the tumor had positive immunoreactivity to desmin, vimentin and smooth muscle actin (SMA). On the basis of immunohistopathology, the tumor was reported as a leiomyoma. Five years later, the lesion recurred at the identical site. An excisional biopsy was performed, and the report again was consistent with leiomyoma with positive immunoreactivity to desmin, vimentin and SMA. An excisional biopsy was done via the transcranial route, and the tumor was reported as leiomyoma. Five years later, the lesion recurred at the identical site. An excisional biopsy was performed, and the report again was consistent with leiomyoma. Now, as the patient presented, his physical examination was remarkable for a palpable 8×9cm, fixed, non-tender, non-ulcerated, nodular mass attached to the underlying structures. The overlying skin was erythematous with prominent and dilated superficial veins.

An ocular examination of the right eye revealed a compressed eyeball, congested conjunctiva and restricted eye movement with complete vision loss. The left eye was normal, with 6/6 visual acuity.

**Figure 2:**
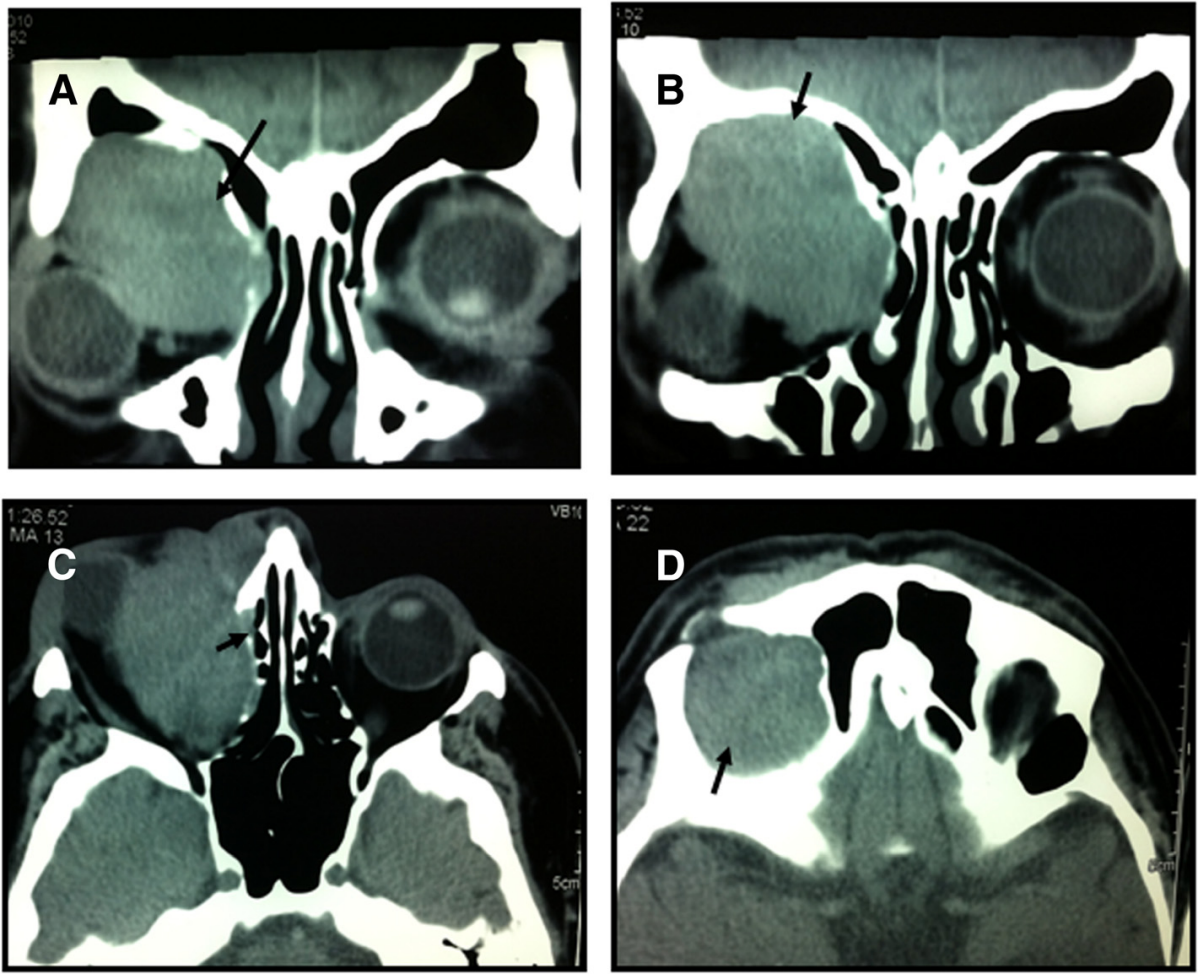
Orbital computed tomographic scans revealing dermatofibrosarcoma protuberans**. (A)** and **(B)** Coronal images. **(C)** and **(D)** Axial images. These computed tomographic scans reveal a well-defined soft tissue mass along the superomedial aspect of the right orbit (arrows in **A** and **B**). The mass is causing pressure remodeling of the right lamina papyracea and ensuing fractional annihilation of the right ethmoidal sinus (arrow in **C**). The mass is also causing pressure remodeling of the right orbital roof, extending cranially (arrow in **D**).

CT of the brain demonstrated mild white matter ischemic changes, and CT of the orbit showed a well-defined, lobulated soft tissue mass along the superomedial aspect of the orbit measuring 5.3×4.8cm and extending 4.3cm craniocaudally. The lesion displaced the eyeball inferolaterally and had both extraconal and intraconal components involving the medial rectus muscle and abutting the optic disc. Medially, partial obliteration of the right ethmoidal sinus due to compression remodeling of the right lamina papyracea was observed. Pressure remodeling of the superior orbital roof was also observed (Figure 2).

After all aseptic measures were done, the tumor was removed *en masse*. A right subfrontal incision was made through the skin, and the supraorbital ridge was exposed. A wide craniectomy was performed with a 6cm rim on the frontal scalp, allowing the tumor to be resected *en bloc*, leaving negative margins. The tumor had eroded through the roof and floor of the orbit superoinferiorly and involved the maxillary sinus inferiorly. The tumor was transected off the right eyeball, which was posteriorly present and necrotized and compressed by the tumor. The entire eyeball was removed, along with the optic nerve and muscles, and a mega-maxillary antrostomy was created. The resulting cavity was filled with oxidized cellulose to prevent hemorrhage. A pericranial autograft was performed. The defect was reconstructed using a titanium mesh plate over the supraorbital part of skull.

Macroscopic inspection of the excised tumor revealed a firm, homogeneous, tannish brown specimen measuring 12cm×13cm×15cm in aggregate. The cut surface was fleshy and homogeneous and had no areas of hemorrhage. Microscopically, the tumor comprised spindle cells with mild nuclear atypia and a low mitotic index. There was scant cytoplasm and an inconspicuous vasculature. The tumor cells were seen to infiltrate around the fat cells, making lacelike patterns. The lesion was positive for CD34 and BCL2 and negative for epithelial membrane antigen (EMA) and cytokeratin 19. On the basis of the immunohistopathology, the tumor was diagnosed as a DFSP.

Postoperative CT (plain and contrast-enhanced) revealed a residual soft tissue mass infiltrating the roof of the maxillary sinus, right-sided ethmoidal air cells and the right nasal bone. Posteriorly, the lesion extended to the superior orbital fissure, causing erosive changes. The infiltrated soft tissue showed post-contrast enhancement.

Following the diagnosis, the patient was started on imatinib mesylate therapy (800mg/day). An adjuvant therapy was advised, and the patient subsequently received radiation therapy at a total dose of 60Gy at 1.8Gy to 2.0Gy per fraction, 5 days per week. The patient’s general condition was very good at the last follow-up visit, 24 months after the surgery. No evidence of local relapse was seen, though there was a residual deformity at the operation site. No enlargement of cervical and axillary lymph nodes was present. The patient continues to be symptom-free with no signs of tumor recurrence.

## Discussion

In accordance with the World Health Organization classification system, DFSP is a locally aggressive mesenchymal tumor that rarely metastasizes, and has intermediate malignancy [8].

DFSP presents with a specific reciprocal translocation at t(17;22)(q22;q13) and supernumerary ring chromosomes derived from t(17;22), leading to the fusion of genes collagen type I, alpha 1 (*COL1A1*), and platelet-derived growth factor beta-chain [9] (*PDGFΒ*).

Furthermore, studies have reported cultured cells of DFSP to be sensitive to treatment with PDGF receptor inhibitors, leading to a decreased rate of cell proliferation [10]. Also, a clinical trial with imatinib showed significant results in cases of DFSP, thus sustaining the position of PDGFB signaling in the development of DFSP [11].

However, to the best of our knowledge, our case is the first of its kind where DFSP was followed by recurrent leiomyoma. Sanz-Trelles and colleagues reported four cases of DFSP in Spain with an unusual morphological change of intraneoplastic blood vessels, where smooth muscles of the vascular wall underwent focal proliferation, leading to decreased luminal diameter. It was so severe in three cases that leiomyomatous nodules and bundles were formed [12]. Thus, we propose in our case that the changes in the microvasculature in the right orbital cavity due to leiomyoma induced the formation of DFSP in the same orbit. A more detailed understanding of the dense factors that regulate the interactions between tumor and surrounding vascular compartments will help us analyze if there is any such possibility where a tumor can induce the development of another neoplastic tissue in its surroundings.

Leiomyomas are characterized as a benign tumor of the smooth muscle. They can manifest in any part of the body, such as the uterus, esophagus and small bowel, and very rarely in the cranial cavity. Uterine leiomyomata are the most prevalent pelvic tumors in women of reproductive age [13]. It has been reported that estrogen and progesterone exhibit important roles in the propagation of uterine leiomyoma [14]. Sozen and Arici elaborated the autocrine and paracrine signaling of the estrogen and progesterone growth factors at the molecular level, which provided insight about the mitogenesis in leiomyoma cells [15]. The growth repression exerted by estrogen is associated with the downregulation of PDGF and mitogen-activated protein kinase in *TSC2*-expressing cells [16]. PDGF is conserved by eight cysteine residues. PDGF receptor isoforms (α-PDGFR and β-PDGFR) activate a downstream signaling cascade by activating receptor tyrosine kinases [17]. It has previously been reported that PDGFs and their receptors are highly expressed in both leiomyoma and myometrial smooth muscle tissue [18].

PDGF acts as a mitogen for mesenchymal cells and plays a pertinent role in the generation of carcinomas [19]. It has been observed that vascular endothelial growth factor, basic fibroblast growth factor and PDGF are implicated in the progression of leiomyomas by augmenting the mitotic activity [20]. The decreased PDGF production in leiomyomas after gonadotropin-releasing hormone analogue treatment causes greater depreciation in uterine volume, implicating that PDGF might have a mitogenic action on leiomyomas [21]. The association of *PDGFB* gene with the orbital neoplasm has been reported [22]. Thus, we propose that the intracranial leiomyoma in our patient acted and progressed by the same mechanism as uterine leiomyomas.

Moreover, it has been reported that eukaryotic genome forms dynamic physical communication with itself in the form of chromosome loops and bridges, which can contribute to the silencing of genes regulating DFSP tumor [23] because the leiomyoma has already enriched the microenvironment with excessive amounts of PDGF. Because DFSPs enclose the receptor for PDGF, we propose that PDGF acts in an autocrine manner to cause cell division and may lead to the development of DFSP. This would account for the existence of tumor markers CD34 and CD56 in both DFSPs and vascular leiomyomas.

Surgical excision is the therapeutic choice in patients with DFSPs [24]. Mohs micrographic surgery is favored because it preserves cosmesis [25]. However, in case of residual tissues, radiation therapy is considered effective. Ballo and colleagues suggested radiation doses of 50Gy to 60Gy as adjuvant therapy to resection if residual tissue is present and proved DFSP to be a radioresponsive tumor [24].

## Conclusions

The hypothesis that a tumor may induce the formation of another kind of tumor in the surrounding tissues cannot be disregarded. The possibility must be evaluated through controlled trials to seek better treatment strategies to prevent such transformations. One potential intervention is the effective utilization of PDGF receptor kinase inhibitors to prevent transformation to any PDGF-driven tumor, which in our patient was DFSP.

## Consent

Written informed consent was obtained from the patient for publication of this case report and any accompanying images. A copy of the written consent is available for review by the Editor-in-Chief of this journal.

## Abbreviations

DFSP: Dermatofibrosarcoma protuberans
*COL1A1*: Collagen, type I, alpha 1 gene
CT: Computed tomography
EMA: Epithelial membrane antigen
PDGF: Platelet-derived growth factor
*PDGFB*: Platelet-derived growth factor beta-chain gene
PDGFR: Platelet-derived growth factor receptor
SMA: Smooth muscle actin
